# Awareness and Attitude Towards Opioid and Stimulant Use and Lifetime Prevalence of the Drugs: A Study in 5 Large Cities of Iran

**DOI:** 10.15171/ijhpm.2018.128

**Published:** 2018-12-31

**Authors:** Elham Mohebbi, Ali Akbar Haghdoost, Alireza Noroozi, Hossein Molavi Vardanjani, Ahmad Hajebi, Roya Nikbakht, Maryam Mehrabi, Akram Jabbarinejad Kermani, Mahshid Salemianpour, Mohammad Reza Baneshi

**Affiliations:** ^1^HIV/STI Surveillance Research Center, and WHO Collaborating Center for HIV Surveillance, Institute for Futures Studies in Health, Kerman University of Medical Sciences, Kerman, Iran.; ^2^Modeling in Health Research Center, Institute for Futures Studies in Health, Kerman University of Medical Sciences, Kerman, Iran.; ^3^School of Advanced Technologies in Medicine (SATiM), Tehran University of Medical Sciences (TUMS), Tehran, Iran.; ^4^Iranian National Center for Addiction Studies (INCAS), Tehran University of Medical Sciences (TUMS), Tehran, Iran.; ^5^MPH Department, Medical School, Shiraz University of Medical Sciences, Shiraz, Iran.; ^6^Research Center for Addiction & Risky Behavior (ReCARB), Psychiatric Department, Iran University of Medical Sciences, Tehran, Iran.; ^7^Department of Sociology, Faculty of Social Sciences and Economics, Alzahra University, Tehran, Iran.; ^8^Department of Mental, Social Health and Drug Use, Ministry of Health, Tehran, Iran.; ^9^Social Determinants of Health Research Center, Institute for Futures Studies in Health, Kerman University of Medical Sciences, Kerman, Iran.

**Keywords:** Cognition, Attitude, Prevalence, Opioid Related Disorders, Amphetamines

## Abstract

**Background:** Providing population-based data on awareness, attitude and practice of drug and stimulant use has policy implications. A national study was conducted among Iranian general population to explore life time prevalence, awareness and attitudes toward opioids and stimulant use.

**Methods:** We recruited subjects from 5 provinces with heterogenic pattern of drug use. Participants were selected using stratified multistage cluster sampling. Data were collected using a validated self-administered questionnaire. Logistic regression model was applied to identify the variables that are associated with drug and stimulant use.

**Results:** In total 2065 respondents including 1155 men (33.96 ± 10.40 years old) and 910 women (35.45 ± 12.21 years old) were recruited. Two-third of respondents had good awareness about adverse effects of opioid use. Corresponding figure in terms of stimulants was 81.4%. Almost 95% of participants reported a negative attitude towards either opioid or stimulant use. The lifetime prevalence of opioid use and stimulant use were 12.9% (men: 21.5%, women: 4.0%) and 7.3% (men: 9.6%, women: 4.9%), respectively. Gender (adjusted odds ratio [AOR]_M/W_=6.92; 95% CI: 2.92, 16.42), education (AOR_undergraduate/diploma or less_ =0.49; 95% CI: 0.26, 0.90), and marital status (AO_Rothers/single_=2.13; 95% CI: 1.36, 3.33) were significantly related with opioid use. With respect to stimulant use, age was negatively associated with the outcome (AOR_60+/20-29 years_ = 0.08: 95% CI; 0.01, 0.98) and men were 2 times more likely than women to use stimulants (OR_M/W_=2.15: 95% CI: 0.83, 5.56). In addition, marital status (AOR_Others/singles_ = 3.45; 95% CI: 1.09, 10.93), and awareness (AOR_Weak and moderate/good_ =0.40; 95% CI: 0.25, 0.61) were independently correlated with stimulants use.

**Conclusion:** While the attitude of Iranian adults toward opioid and stimulant use was negative, their awareness was not that adequate to prevent the drug use. Men and those with lower socio-economic status (SES) should be the focus of health promotion programs regarding opioid use. However, regarding stimulants use, promotion programs should target younger age groups and those with higher SES status.

## Introduction

Illicit drug use poses striking challenges to health systems worldwide. In 2016, 275 million of world population aged 15-64 used an illicit drug, which was accounted for 5.6% of the population.^1^ In addition to that, global mortality and morbidity attributed to illicit drug use, which mostly happened in Eastern Europe and Central Asia, is substantial (37 926 000 million disability-adjusted life years [DALYs]). Opioid dependence was the largest contributor of years life lost (YLLs) to premature mortality for illicit drug use disorders.^2^ In 2010, global burden of diseases estimated 804.5 and 227 for DALYs and 351.8 and 24.8 for YLLs in Iranian men and women, respectively.^3^

Several studies confirmed that increase in drug use disorders are associated with increase in number of shattered families, as well as crime, violence and insecurity, and health related problems including HIV and hepatitis C (via injecting drugs).^4,5^ It also affects economic costs, especially in low- and middle-income countries (LMICs).^6^

Iran is an Islamic middle-income country with a population of more than 80 million, of whom more than 70% live in urban settings.^7^ Except for medical purposes, using opioids and stimulants are illegal in Iran. Despite negative attitudes toward drug use among older Iranians, it has been less stigmatized and even partly prestigious among youths.^8-10^ This could make drug-related issues uncontrollable especially among younger adults and women.^6^ According to the national household survey, last year prevalence of any illicit drug use disorder except alcohol was 2.44% (1.56% drug dependence), accounting for approximately 1.12 million Iranians (15-64 years). Amongst the illicit drugs, opioids were the most prevalent one with prevalence of 2.23% (95% CI; 1.83%, 2.62%), following by cannabis (0.56%) and amphetamine-type stimulants (ATS) (0.39%).^8^ Moreover, prevalence of ATS used is very high in specific sub-population, for instance 73.9% among transit driver and 46.5%.^11,12^

Opioids are the most popular drug of use in Iran drug scene and have been used more than 5 centuries, partly due to long and porous border with Afghanistan, the main producer of illicit opioids in the world.^2,13^ In contrast, ATS are almost the new brand drugs in Iran’s market (since 2005),^14^ firstly, imported from southeastern border. While ATS use is associated with many mental (insomnia, anxiety, and psychosis) and physical health complications (increased body temperature, and increased rapid heart rate), knowledge about its side effects is low.^15-17^ Besides, international sanctions negatively affect economic growth which might result in social harms including illicit drug use.^18^

There are several references to opium – “Tariak” in Persian – in Iranian medieval and classic poems where opium was considered as an “antidote” to treat diseases. Furthermore, opium has been used to treat pain, cough and diarrhea in Iranian traditional medicine.^19^ Nowadays, patients adhere to the faulty superstition about opium and sometimes initiating to use it by disease development without any awareness about the side effects and addictive properties.^17,20^ Available evidences on awareness and attitude toward opium are slim and the focuses of majority of such studies are on nursing and medical students, or high school students.^21-23^

Although ATS are the new brand drug in Iran illicit drug markets, a number of studies – including Persian and English and gray literature – evaluated amphetamine and ecstasy use awareness and attitude. Nonetheless, results of the most recent knowledge, attitude and practice study on Iranian youths (4868 participants aged 15-29 years) showed that 42% of participants had low knowledge, and about one-third of them had positive attitudes towards amphetamine use.^24^

As mentioned above majority of studies were concentrated on specific groups. However, we believe that general population should be the main target group for health education and promotion strategies. Therefore, a large study was conducted to explore life time prevalence of opioid and stimulant use, as well as awareness and attitudes toward opioid and stimulant use among Iranian adults.

## Methods

### Participants and Sampling Design

In 2013, Iranian National Network Scale Up (NSU) study was designed to estimate the prevalence of opioids and to stratify the country into low, intermediate, and high-risk zones. At the first step, results of that study were used to select 5 provinces: West Azerbaijan and Markazi from low risk stratum, Tehran and Khorasan-e-Razavi from moderate risk stratum, and Kerman from high risk stratum. Data are collected in capitals. Each city was stratified into low, middle and high socio-economic zones.

A street-based consecutive sampling was used to recruit the respondents at different times and locations. The inclusion criteria were consent verbally obtained, over 18 years old, and able to reading and writing in Persian.

Sample size calculation was based on information obtained from evaluating drug abuse awareness among Afghans (88.6%).^25^ We assumed that proportion of awareness about drug use 0.9 and d = 0.03 in both sexes ($n=\frac{z^{2}p(1-p)}{d^{2}}$). This suggests a sample size of 400 for each province.

### Designing Instrument

A self-administered questionnaire was designed considering socio-cultural context of Iran. The questionnaire was developed in 4 phases; (1) literature review, (2) expert panel review, (3) focus-group testing and evaluation, and (4) and reliability and validity assessment.

An extended literature review was conducted to ascertain common awareness and attitude statements related to opioid and stimulant use, and to find available instruments. We found various studies and reports.^26-33^ Reviewing the questionnaires, a question pool inquiring awareness, attitude and behavior on opioid and stimulants use was developed and discussed in expert panel reviews.

### Expert Panel Review

Experts from Iranian National Center for Addiction Studies (INCAS) [Tehran, Iran], Tehran University of Medical Sciences (TUMS) [Tehran, Iran], and Neuroscience Research Center, Institute of Neuropharmacology [Kerman, Iran] reviewed the questionnaires and their invaluable feedbacks were gathered.

### Focus Group Discussion

We conducted 2 focus group discussions (FGDs) to explore attitudes and opinions of youth population with respect to common type of drugs used among Iranian adults. A convenient sample of university students (n = 15) were selected from Kerman University of Medical Sciences (KUMS), Kerman, Iran. A letter was sent to these students explaining the purpose of the focus groups and assuring them that confidentiality would be maintained. In the first session, attitudes, perceived reasons and contexts of drug use initiation were discussed. In the second session the participants’ opinions regarding most prevalent type of drugs were explored and then participants were asked to talk about their awareness, attitude and experiences about drug use. The focus groups were facilitated by EM.

### Designing of the Questionnaire

Based on lessons from FGD, 6 conceptual frameworks were drawn for awareness, attitude and life time prevalence of opium and stimulant use. Draft was revised several times according to the experts’ feedback so that the face validity of final version was approved by experts and consultants.

### Assessment of Reliability and Validity

To address the validity, we asked 34 medical doctors and nurses, who worked in medical centers, to fill the questionnaire. Cronbach α was calculated at 0.90 for awareness, 83% for attitude, and 87% for practice.

For validity assessment, we asked 5 experts to describe the relevance degree of the questions (completely relevant, relatively relevant, relevant, and irrelevant). Content validity index (CVI) was estimated at about 0.8 which confirmed that validity of our questionnaire.

### Structure of Questionnaire

In the first part of the questionnaire, using 20 questions with 3 possible answers (correct, incorrect, and I do not know), awareness towards opioid and stimulants was assessed. Based on the number of correct replies, 3 categories were defined, weak (0 to 4), moderate (5 to 7), and good awareness (8 to 10).

In the second section, which was consisted of 24 five-point Likert scale questions, we measured the attitude. The questions were associated with attitude toward social, cultural, legalization, and religious contexts. An overall attitude was determined by aggregating all positive responses ranging from -24 to +24. Four categories were defined as: (I) strongly disagree [-24 to -13]; (II) disagree [-12 to -1]; (III) agree [+1 to +12]; and (IV) strongly agree [+13 to +24].

The third part was devoted to practice of opioids and stimulants. We asked participants “Have you ever used opioid or stimulant, even one episode, during your life?” Opioids included opium, opium dross, condensed opium, heroin, crack-heroin, methadone, tramadol, and codeine. Stimulants included crystal methamphetamine, amphetamine, ecstasy, cocaine and Ritalin. Examples of drugs in each class and their common street names were provided in the questionnaire. In the last part of the questionnaire, demographic information was collected.

### Data Collection Methods and Instrument

Data were collected by using the self-administered questionnaire along with guidance provided by interviewers in 2015. At the first step, each city was stratified into low, middle and high socio-economic zones. A street-based consecutive sampling was used to recruit the respondents adopting time location sampling (TLS). We randomly selected some streets at different zones and asked data collectors to collect the data at different times. We asked them to recruit participants with different clothing and appearance to maximize the generalizability. Only pedestrians who walked alone were approached.

After taking verbal consent, interviewees were asked to complete study questionnaire anonymously in the street corners. We matched the gender of data collector and interviewee. Interviewer took about 2-3 m distance from the respondent to avoid violation of the participants’ privacy. However, study participants’ questions were answered by the interviewers. An opaque envelope was provided for putting the questionnaire among completed questionnaires by other participants.

### Data Manipulation and Statistical Analysis

Data was cleaned and prepared based on the study protocol. Variables with more than 40% missing data were excluded. For the rest of variables, missing data were imputed applying multiple imputation by chained equations (MICE) method 10 times. Linear, logistic, and multinomial regression models were used to impute missing data for linear, binary, and multinomial variables.

Chi-square test and independent samples *t* test were used to determine the associations between qualitative and quantitative variables with opioid and stimulants use, respectively. *P* values less than 5% were considered as statistically significant. Logistic regression was applied to assess the association of demographic characteristics, awareness, and attitude with life time prevalence of opioid and stimulant use. Appropriate weights were applied, with respect to age and gender, to adjust for our sampling scheme. All variables were offered to the model. Using each imputed data set, a logistic regression model was fitted. Results of 10 models were combined applying Rubin Rules. The variable with the highest *P* value was excluded. The process continued until all *P* values remained below 0.05. This iterative process guarantees exclusion of unimportant and correlated variables. We entered variables in the model that had missing data less than 40% and backward model was performed. Analyses were done using Stata v.12 (Stata Corporation College Station, TX, USA) and R software.

## Results

The response rate was 86.0%. A total of 2065 individuals (1155 men and 910 women) filled the questionnaire. The mean age of men and women were 33.96 ± 10.40 and 35.45 ± 12.21 respectively. More than 70% of participants had an undergraduate academic degree. Self-reported monthly income for almost 65% of participants was less than $334 (exchange rate 1$: 4200 IRR) (Table 1).

**Table 1:** Demographic and Socioeconomic Characteristics of Participants

**Variable**	**Men (n = 1155)**	**Women (n = 910)**	***P***	**Total (N = 2065)**
Age (y)	33.96 ± 10.40	35.45 ± 12.21	.004	34.80 ± 11.47
Marital status, No. (%)			<.0001	
Never married	419 (36.3)	413 (45.4)		832 (40.3)
Married	644 (55.8)	392 (43.1)		1036 (50.2)
Others	92 (8.0)	105 (11.5)		197 (9.5)
Education, No. (%)			.395	
High school or less	292 (25.3)	212 (23.3)		504 (24.4)
Undergraduate	813 (70.4)	650 (71.4)		1463 (70.8)
Graduate	50 (4.3)	48 (5.3)		98 (4.7)
Income (US$), No. (%)			<.0001	
Less than 167	304 (26.3)	418 (45.9)		772 (35.0)
167-334	358 (31.0)	287 (31.5)		645 (31.2)
334-668	399 (34.5)	173 (19.0)		572 (27.7)
More than 668	94 (8.1)	32 (3.5)	126 (6.1)
Employment status, No. (%)		<.0001	
Unemployed	335 (29.0)		554 (60.9)	889 (43.1)
Employed	820 (71.0)		356 (39.1)	1176 (56.9)

### Awareness

#### Opioid Use

Most of participants had good awareness about adverse effects of opioids on health (66.94%, 95% CI: 54.4, 79.5) and only 1.5% were not aware of adverse effects (95% CI: 0.0, 1.5). About one-third of respondents had moderate level of awareness (31.5%, 95% CI: 20.4, 42.6).

Higher education was positively correlated with higher awareness. While 75% (95% CI: 69.5, 80.9) of those with graduate degree had good awareness, corresponding figure among those with high school or lower degree was 60.1% (95% CI: 38.1, 82.1). Marital status (*P* = .09) and employment status (*P* = .58) and age (*P* = .17) of respondents did not show association with their awareness but income (*P* < .0001) (Table 2).

**Table 2:** Awareness of Iranian Adults Towards Opioid Use, 2015

	**Variable**		**Knowledge (%, 95% CI)**			
			**Good**	**Moderate**	**Weak**	***P***
Opioids	Gender	Men	63.3 (49.0, 77.7)	35.0 (22.4, 47.6)	1.6 (0.0, 3.5)	<.0001
		Women	70.8 (50.2, 91.2)	27.9 (9.6, 46.2)	1.4 (0.0, 3.6)	
	Education	Diploma or less	60.1 (38.1, 82.1)	37.6 (16.6, 58.6)	2.2 (0.0, 3.8)	<.0001
		Undergraduate	69.0 (61.7, 76.3)	29.6 (23.6, 35.6)	1.3 (0.0, 2.9)	
		Graduate	75.2 (69.5, 80.9)	24.5 (19.1, 30.5)	-	
	Age	20-29	66.3 (54.4, 78.2)	32.6 (22.1, 43.0)	1.1 (0.0, 2.6)	.165
		30-39	65.4 (55.6, 75.2)	32.9 (25.0, 40.8)	1.7 (0.0, 3.7)	
		40-49	67.2 (51.4, 82.9)	30.8 (16.7, 45.0)	2.0 (0.0, 4.7)	
		50-59	70.8 (52.1, 82.9)	26.8 (8.7, 45.0)	2.4 (0.0, 4.6)	
		More than 60	69.9 (48.0, 91.8)	30.0 (8.2, 52.0)	-	
	Income	Less than 167	60.8 (42.0, 79.6)	37.0 (19.6, 54.3)	2.2 (0.0, 3.9)	<.0001
		167-334	68.3 (60.5, 76.2)	30.0 (24.2, 35.9)	1.6 (0.0, 3.9)	
		334-668	74.2 (64.3, 84.0)	25.4 (16.1, 34.8)	0.3 (0.0, 1.1)	
		More than 668	68.7 (63.9, 73.5)	30.0 (24.9, 35.3)	1.2 (0.0, 2.7)	
	Marital status	Never married	68.6 (57.6, 79.6)	30.1 (19.9, 40.4)	1.2 (0.0, 2.1)	.085
		Married	67.7 (53.1, 82.3)	30.5 (17.7, 43.3)	1.7 (0.0, 3.7)	
		Others	60.0 (45.4, 74.5)	38.6 (25.5, 51.7)	1.4 (0.0, 3.7)	
	Employment status	Unemployed	65.5 (45.0, 86.1)	33.1 (14.1, 52.0)	1.4 (0.0, 3.0)	.579
		Employed	68.2 (62.7, 73.7)	30.2 (25.9, 34.4)	1.6 (0.0, 3.2)	
	**Total**		**66.94 (54.4, 79.5)**	**31.5 (20.4, 42.6)**	**1.5 (0.0, 3.0)**	

#### Stimulant use

Almost 81.4% (95% CI: 76.7, 86.2) of participants had good awareness about adverse effects of stimulants on health and only 3% (95% CI: 1.2, 4.8) had weak awareness (Table 3).

**Table 3:** Awareness of Iranian Adults Towards Stimulant Use, 2015

	**Variable**		**Knowledge (%, 95% CI)**			
			**Good**	**Moderate**	**Weak**	***P***
Stimulants	Gender	Men	80.7 (71.4, 85.4)	15.6 (7.3, 17.8)	3.7 (1.2, 6.1)	<.0001
		Women	82.2 (79.0, 85.4)	15.5 (13.1, 17.8)	2.3 (0.0, 4.6)	
	Education	Diploma or less	73.7 (68.1, 79.4)	21.2 (13.6, 28.7)	5.1 (2.3, 7.8)	<.0001
		Undergraduate	83.8 (76.9, 90.8)	13.8 (7.5, 20.0)	2.4 (0.0, 3.8)	
		Graduate	90.3 (85.1, 95.5)	9.2 (4.8, 13.8)	3.8 (0.0, 1.4)	
	Age	20-29	80.5 (75.1, 85.9)	16.7 (11.3, 22.0)	2.8 (1.6, 4.0)	.006
		30-39	83.1 (77.6, 88.6)	12.9 (7.4, 18.4)	4.0 (0.0, 7.5)	
		40-49	79.5 (71.8, 87.0)	16.9 (11.3, 22.4)	3.7 (0.0, 7.2)	
		50-59	83.2 (77.2, 89.3)	15.6 (10.1, 21.0)	1.1 (0.0, 3.0)	
		More than 60	81.9 (58.2, 100)	16.8 (0.0, 41.4)	1.2 (0.0, 2.8)	
	Income	Less than 167	79.0 (76.0, 82.0)	15.7 (12.2, 19.2)	5.3 (2.8, 7.8)	.0009
		167-334	81.5 (76.1, 86.9)	16.0 (12.0, 20.0)	2.5 (0.0, 4.6)	
		334-668	82.1 (72.2, 91.9)	17.0 (7.6, 26.4)	0.9 (0.0, 2.3)	
		More than 668	91.0 (82.5, 99.5)	8.5 (0.0, 16.5)	0.4 (0.0, 1.5)	
	Marital status	Never married	81.3 (74.7, 87.9)	15.8 (8.7, 22.9)	2.8 (1.4, 4.2)	0.028
		Married	82.8 (77.0, 88.6)	14.2 (9.3, 19.0)	3.0 (0.0, 6.2)	
		Others	76.7 (68.9, 84.4)	19.8 (9.8, 29.9)	3.4 (0.0, 6.5)	
	Employment status	Unemployed	81.4 (76.7, 86.2)	15.1 (10.8, 19.3)	3.5 (1.8, 5.1)	.888
		Employed	81.4 (76.2, 86.7)	16.0 (10.7, 21.3)	2.5 (0.0, 4.9)	
	**Total**		**81.4 (76.7, 86.2)**	**15.5 (11.2, 20.0)**	**3.0 (1.2, 4.8)**	

Higher education (*P* < .0001) and monthly income (*P* = .009) were positively correlated with higher awareness. There was association between gender and awareness (*P* < .0001), age (*P* = .006), and marital status (*P* = .028) with outcome. Employment status (*P* = .888) showed no association with respondents’ awareness (Table 3).

### Attitude

#### Opioid Use

95.5% (95% CI: 91.5, 99.5) of participants reported a negative attitude toward opioid use. Higher education was the only variable which was significantly correlated with attitude (*P* = .02) (Table 4). While 7.4% of respondents with low education reported a positive attitude, corresponding figure among graduated respondents was 1.2%.

**Table 4:** Attitude of Iranian Adults Towards Opioid and Stimulant Use, 2015

**Variable**		**Opioids (n = 2065)**			**Stimulants (n = 2065)**		
		**Negative**	**Positive**	***P***	**Negative**	**Positive**	***P***
Gender	Men	94.5 (88.4, 100)	5.5 (0.0, 11.6)	.55	97.1 (93.0, 100)	2.9 (0.0, 7.0)	.72
	Women	96.6 (91.8, 100)	3.4 (0.0, 8.2)		96.2 (92.2, 100)	3.9 (0.0, 7.9)	
Education	High school or less	92.6 (88.2, 97.0)	7.4 (3.0, 11.8)	.02	95. 1 (92.8, 7.4)	4.9 (2.6, 7.2)	.05
	Undergraduate	96.4 (93.0, 99.9)	3.6 (0.1, 7.0)		96.9 (93.8, 100)	3.1 (0.0, 6.2)	
	Graduated	98.8 (96.0, 100)	1.2 (0.0, 4.0)		99.6 (98.5, 100)	0.4 (0.0, 1.5)	
Age	20-29	95.3 (0.9, 99.9)	4.7 (0.06, 9.3)	.37	96.0 (91.9, 100)	4.0 (0.0, 8.1)	.32
	30-39	96.2 (0.9, 98.6)	3.8 (1.4, 6.3)		97.0 (94.8, 99.3)	3.0 (0.7, 5.2)	
	40-49	96.8 (91.3, 100)	3.2 (0.0, 8.7)		98.0 (95.4, 100)	2.0 (0.0, 4.6)	
	50-59	93.5 (87.7, 99.2)	6.6 (0.7, 12.3)		95.3 (91.3, 99.4)	4.7 (0.6, 8.8)	
	More than 60	93.9 (87.2, 100)	6.1 (0.0, 12.8)		96.8 (92.4, 100)	3.2 (0.0, 7.6)	
Income	Less than 167	94.2 (89.7, 98.6)	5.8 (1.4, 10.3)	.16	95.8 (92.8, 98.9)	4.2 (1.2, 7.2)	.19
	167-334	96.4 (92.7, 100)	3.6 (0.0, 7.2)		97.0 (93.8, 100)	3.1 (0.0, 6.2)	
	334-668	95.7 (92.3, 99.1)	4.3 (0.9, 7.7)		96.7(93.8, 99.5)	3.3 (0.5, 6.2)	
	More than 668	97.8 (92.9, 100)	2.2 (0.0, 7.1)		99.0 (96.5, 100)	1.0 (0.0, 3.5)	
Marital status	Never married	96.0 (92.5, 99.5)	4.0 (0.5,7.5)	.6	97.1 (94.3, 99.9)	2.9 (0.1, 5.7)	.06
	Married	94.7 (90.3, 99.0)	5.3 (1.0, 9.7)		96.1 (93.2, 98.9)	3.9 (1.0, 6.8)	
	Others	97.4 (94.3, 100)	2.6 (0.0, 5.7)		97.5 (94.7, 100)	2.5 (0.0, 5.3)	
Employment status	Unemployed	94.7 (90.3, 99.1)	5.3 (0.9, 9.7)	.24	95.6 (92.2, 99.0)	4.4 (1.0, 99.0)	.11
	Employed	96.2 (92.3, 100)	3.8 (0.0, 7.7)		97.5 (94.8, 100)	2.5 (0.0, 5.2)	
**Total**		**95.5 (91.5, 99.5)**	**4.5 (0.5, 8.5)**		**96.6 (93.7, 99.5)**	**3.4 (0.5, 6.3)**	

#### Stimulant Use

More than 96.0% (95% CI: 93.7, 99.5) of respondents had a negative attitude towards stimulants. Almost all of respondents (99.6, 95% CI: 98.5, 100) with a graduate degree reported a negative attitude regarding stimulants consumption. Negative attitude among less educated respondents was 95.1 (95% CI: 92.8, 97.4; *P* = .05). Marital status was marginally associated with attitude (*P* = .06). Negative attitude in men and women were estimated at 97.1% and 96.2% respectively (*P* = .72). Age (*P* = .32), monthly income (*P* = .19) and employment status (*P* = .11) had no significant association with attitude toward stimulants use (Table 4).

### Life Time Prevalence

### Opioid Use

The self-reported life time prevalence of opioid use was estimated at 12.9% (95% CI: 6.8, 19.0) (Table 5). Lifetime prevalence of opioid use was significantly higher in men than women — 21.5% (95% CI: 18.7, 24.3) versus 4.0% (95% CI: 1.9, 6.1). Prevalence in those aged >60 and 40-49 were 20.9% (95% CI: 0.0, 43.4%), and 19.3% (95% CI: 8.8, 29.7) respectively. With respect to the marital status, the highest prevalence was estimated for whom categorized as “others,” including divorced, widows and separated (20.5, 95% CI: 10.1, 30.9). Respondents with lowest education (19.8%, 95% CI: 5.0, 34.5) and who were employed (15.7% 95% CI: 9.7, 21.8) reported highest lifetime prevalence of opioid use. With respect to income, highest prevalence was observed among middle lower incomes ($167-$334 per month) (Table 5).

**Table 5:** Lifetime Prevalence (95% CI) of Opioid Use Amongst Iranian Adults

**Type of Drugs**	**Variables**	**Men (n = 1155)**	**Women (n = 910)**	***P***	**Total (n = 2065)**
Opioids	Age				
	20-29	17.2 (14.4, 19.9)	4.6 (2.1, 7.0)	.038	10.8 (7.0, 14.6)
	30-39	18.7 (7.3, 30.2)	3.8 (0.0, 7.6)	.028	11.0 (4.8, 17.2)
	40-49	30.7 (21.3, 40.1)	6.3 (2.2, 10.5)	<.0001	19.3 (8.8, 29.7)
	50-60	18.8 (15.0, 22.5)	0.8 (0.0, 2.7)	.225	10.0 (2.8, 17.3)
	60+	32.4 (15.6, 49.1)	-	-	20.9 (0.0, 43.4)
	Marital Status				
	Never Married	16.5 (12.8, 20.2)	3.7 (2.5, 5.0)	.051	10.3 (5.9, 14.6)
	Married	22.1 (19.2, 24.9)	2.4 (0.0, 6.4)	.028	12.6 (5.4, 19.8)
	Others	31.8 (27.5, 36.1)	10.0 (1.9, 18.1)	<.0001	20.5 (10.1, 30.9)
	Education				
	Diploma or Less	36.1 (29.2, 43.0)	2.5 (0.0, 6.0)	.001	19.8 (5.0, 34.5)
	Undergraduate	15.6 (13.5, 17.6)	5.3 (2.6, 7.9)	.063	10.4 (7.9, 13.0)
	Graduate	13.8 (11.4, 16.0)	-	-	7.5 (0.0, 17.4)
	Income (US$)				
	Less than 167	23.1 (13.2, 33.0)	2.4 (0.0, 4.7)	.023	9.5 (1.4, 17.7)
	167-334	24.1 (19.8, 28.4)	7.5 (2.9, 12.0)	.002	16.1 (11.1, 21.1)
	334-668	20.6 (15.5, 25.7)	3.6 (0.0, 10.8)	.018	15.2 (5.6, 24.7)
	More than 668	14.6 (6.2, 23.0)	2.2 (0.0, 9.3)	.123	11.3 (4.5, 18.1)
	Employment status				
	Unemployed	21.2 (17.8, 24.7)	3.0 (1.3, 4.7)	.023	9.8 (2.8, 16.9)
	Employed	21.7 (17.7, 25.7)	5.5 (1.5, 9.4)	.007	15.7 (9.7, 21.8)
**Total**		**21.5 (18.7, 24.3)**	**4.0 (1.9, 6.1)**	**.013**	**12.9 (6.8, 19.0)**

### Stimulant Use

Overall lifetime prevalence of stimulant use was estimated at 7.3% (95% CI: 5.2, 9.4). Estimated prevalence for men was about 2 times that of women — 9.6% (95% CI: 6.1, 13.1) versus 4.9% (95% CI: 0.7, 9.2). Among different age groups, the highest lifetime prevalence was observed among 20-29 years, 12.0% (95% CI: 7.8, 16.2). As the respondents were getting older, the prevalence was sharply declined to 1.7 (95% CI: 0.0, 5.3). With respect to marital status, those categorized as “others” reported the highest prevalence of lifetime stimulant use (13.6, 95% CI: 5.6, 21.6). Those with graduate degree were about 2 times higher than that of those with a degree below diploma (11.9 versus 6.5). Prevalence of stimulant use among those felt into highest monthly income category was noticeable (10.5, 95% CI: 7.0, 14.0) (Table 6).

**Table 6:** Lifetime Prevalence (95% CI) of Stimulants Use Amongst Iranian Adults

**Type of Drugs**	**Variables**	**Men (n = 1155)**	**Women (n = 910)**	***P***	**Total (n = 2065)**
Stimulants	Age				
	20-29	14.7 (11.4, 18.1)	9.4 (0.0, 18.8)	.233	12.0 (7.8, 16.2)
	30-39	8.7 (7.2, 10.1)	3.2 (1.1, 5.4)	.322	5.9 (4.3, 7.4)
	40-49	8.1 (4.6, 11.6)	2.3 (0.0, 4.6)	.349	5.3 (3.2, 7.5)
	50-60	3.2 (0.0, 9.2)	0.4 (0.0, 1.7)	.755	1.9 (0.0, 4.7)
	60+	2.7 (0.0, 8.6)	-	-	1.7 (0.0, 5.3)
	Marital Status				
	Never Married	13.6 (10.0, 17.1)	4.8 (2.5, 7.2)	.109	9.3 (7.6, 10.9)
	Married	5.8 (1.8, 9.8)	2.8 (0.0, 5.0)	.558	4.4 (2.1, 6.6)
	Others	14.5 (3.3, 25.6)	12.8 (0.0, 25.8)	.684	13.6 (5.6, 21.6)
	Education				
	Diploma or Less	12.2 (6.6, 17.8)	0.4 (0.0, 1.0)	.473	6.5 (1.2, 11.8)
	Undergraduate	7.5 (3.8, 11.2)	6.5 (1.9, 11.1)	.818	7.0 (4.3, 9.7)
	Graduate	14.3 (11.6, 17.0)	9.0 (4.5, 13.4)	.237	11.9 (7.3, 16.3)
	Income (US$)				
	Less than 167	15.9 (12.2, 19.6)	2.7 (0.0, 5.5)	.077	7.2 (2.9, 11.6)
	167-334	7.8 (1.2, 14.1)	8.5 (2.6, 14.5)	.869	8.1 (3.4, 12.7)
	334-668	5.5 (2.0, 9.0)	5.8 (3.2, 8.4)	.944	5.6 (3.0, 8.2)
	More than 668	12.1 (6.5, 17.7)	6.0 (3.2, 8.8)	.210	10.5 (7.0, 14.0)
	Employment status				
	Unemployed	11.1 (6.0, 16.3)	4.8 (0.0, 10.3)	.220	7.2 (3.4, 10.9)
	Employed	8.7 (5.9, 11.6)	5.2 (3.0, 7.3)	.456	7.4 (6.1, 8.8)
**Total**		**9.6 (6.1, 13.1)**	**4.9 (0.7, 9.2)**	**.335**	**7.3 (5.2, 9.4)**

### Correlates of Lifetime Prevalence

#### Opioid Use

Results of multivariable logistic regression modeling suggested that gender (AOR _M/F_ = 6.92; 95% CI: 2.92, 16.41), and marital status (AOR_others/single_ = 2.13; 95% CI: 1.36, 3.33) were significantly correlated with lifetime prevalence of opioid use (Table 7). In addition, one level increase in education was associated with 60% decrease in odds of opioids use (*P* < .001).

**Table 7:** Determinants of Opioid Use in Iran

**Type of Drugs**	**Variables**	**Crude OR (95% CI)**	***P***	**Adjusted OR (95%CI)**	***P***
Opioids	Age				
	20-29	Reference			
	30-39	1.02 (0.58, 1.81)	.928	0.97 (0.46, 2.03)	.931
	40-49	1.97 (1.39, 2.79)	.002	1.07 (1.70, 2.89)	.037
	50-59	0.92 (0.51, 1.66)	.762	0.88 (0.49, 1.59)	.629
	60+	2.19 (0.72, 6.60)	.141	1.39 (0.45, 4.24)	.519
	Gender				
	Men	Reference			
	Women	6.58 (3.74, 11.59)	<.0001	6.92 (2.92, 16.42)	<.0001
	Education				
	Diploma or less	Reference			
	Undergraduate	0.47 (0.22, 1.00)	.051	0.49 (0.26, 0.90)	.027
	Graduate	0.33 (0.10, 1.04)	.057	0.31 (0.23, 0.41)	<.001
	Marital status				
	Single	Reference			
	Married	1.26 (0.79, 2,00)	.282	1.06 (0.79, 1.42)	.663
	Others	2.25 (1.42, 3.60)	.004	2.13 (1.36, 3.33)	.004
	Income				
	Less than 167	Reference			
	167-334	1.82 (0.72, 4.62)	.174	1.34 (0.54, 3.29)	.760
	334-668	1.70 (0.63, 4.58)	1.230	1.10 (0.63, 1.91)	.400
	More than 668	1.21 (0.53, 2.74)	.550	0.68 (0.26, 1.78)	.382
	Employment status				
	Unemployed	Reference			
	Employed	1.71 (0.90, 3.24)	.089	1.22 (0.73, 2.04)	.394
	Attitudes				
	Negative	Reference			
	Positive	1.85 (0.57, 6.02)	.263	1.52 (0.58, 3.99)	.347
	Awareness				
	Good	Reference			
	Weak	0.62 (0.21, 1.84)	.341	0.61(0.88, 4.23)	.574
	Moderate	0.30 (0.06, 1.40)	.109	0.34 (0.03, 4.00)	.341

Abbreviation: OR, odds ratio.

#### Stimulant Use

One year increase in age was associated with 7% decrease in odds of stimulants use (*P* = .04). Marital status (AOR_Others/singles_ = 3.45; 95% CI: 1.09, 10.93), and awareness (AOR_Weak and moderate/good_ = 0.40; 95% CI: 0.25, 0.60) had independent correlation with life time prevalence of stimulants use (Table 8).

**Table 8:** Determinants of Stimulants Use in Iran

**Type of Drugs**	**Variables**	**Crude OR (95% CI)**	***P***	**Adjusted OR (95%CI)**	***P***
Stimulants	Age				
	20-29	Reference			
	30-39	0.46 (0.31, 0.68)	.002	0.44 (0.22, 0.89)	.027
	40-49	0.41 (0.25, 0.68)	.004	0.32 (0.14, 0.75)	.015
	50-59	0.14 (0.03, 0.73)	.025	0.11 (0.01, 0.88)	.040
	60+	0.13 (0.01, 1.09)	.058	0.08 (0.01, 0.98)	.049
	Gender				
	Men	Reference			
	Women	2.03 (0.76, 5.44)	.136	2.15 (0.83, 5.56)	.099
	Education				
	Diploma or less	Reference			
	Undergraduate	1.10 (0.42, 2.86)	.220	0.75 (0.35, 1.61)	.410
	Graduate	1.95 (0.68, 5.56)	.180	1.51 (0.73, 3.11)	.219
	Marital status				
	Single	Reference			
	Married	0.44 (0.25, 0.78)	.011	0.86 (0.44, 1.71)	.632
	Others	1.54 (0.70, 3.40)	.245	1.69 (1.10, 10.72)	.037
	Income				
	Less than 167	Reference			
	167-334	1.13 (0.39, 3.27)	.797	1.38 (0.51, 3.76)	.481
	334-668	0.76 (0.31, 1.89)	.512	1.42 (0.62, 3.23)	.356
	More than 668	1.50 (0.71, 3.17)	.242	2.00 (1.32, 3.01)	.005
	Employment status				
	Unemployed	Reference			
	Employed	1.04 (0.60, 1.78)	.877	0.65 (0.41, 1.00)	.052
	Attitudes
	Negative	Reference			
	Positive	1.71 (0.63, 4.61)	.249	1.51 (0.70, 3.23)	.252
	Awareness				
	Good	Reference			
	Weak	0.30 (0.10, 0.85)	.029	0.35 (0.15, 0.60)	.007
	Moderate	0.15 (0.04, 0.50)	.007	0.45 (0.12, 0.83)	.019

Abbreviation: OR, odds ratio.

## Discussion

A large cross-sectional study in 5 major cities of Iran was performed to estimate population-based statistics on the awareness, attitudes towards opioid and stimulant use and their lifetime prevalence. This study reflected that participants had a good level of awareness of opioid (67%) and stimulant use (82%) and almost all of the respondents had negative attitudes toward the drugs. Lifetime prevalence of opioid and stimulant use was 12.9% and 7.2%, respectively.

Good level of awareness observed among Iranian adults in general population was higher than corresponding figures among Iranian students^34,35^; which may be due to lack of comprehensive and mandatory course in educational system, though lower awareness is expected among youths. Moreover, a most recent population-based study on Iranian youths (19-29 years) showed that 42% of them had good knowledge on methamphetamine, which is lower than our findings. We should mention that our questioner was not the same as that used in study.^24^ While we covered all types of stimulants and drugs, they only asked about methamphetamines in details. Some other evidences, related to pre-epidemic phase of stimulants use in Iran, reflected very low knowledge among students(1%-2%).^36,37^ Amphetamine epidemic in Iran happened during 2005-2008 when domestic production of glass was burgeoned. During the epidemic mass media and newspapers abundantly provided documentaries, video clips and articles about adverse effects of stimulant use, therefore, in parallel these have formed negative attitude toward stimulants use.^14,38^

On the other hand, limited evidence on awareness about opioids among Iranian adults is available. Being on the main route of drug trafficking, from Western Asia to Eastern Europe, as well as cultural issues (medical and recreational uses) and relatively low cost, provoke Iranian to use opioids. In contrast having good knowledge about opioid use, colonialism of the Great Britain on Iran opioid market caused negative attitude toward opioid use.^39,40^

While men were more likely to use opioids and stimulants, no association between gender and attitude toward opioids and stimulants use was found. This might be partially justified by previous evidences which suggested that a considerable proportion of people use in their life course drugs before the formation of negative attitudes.^41^

Lifetime prevalence of opioid use reported about 17% in the Northern and southeastern of Iran (ever users), which are known as hot zones and high prevalent areas.^42,43^ Estimation of prevalence of opioid use in specific populations was of great attention. Exploring drug use among high school students indicated wide variation of experiencing opioids, 1.2% to 8.6%.^22^ Some specific populations such as drivers and prisoners were categorized as high risk groups of opioid use, with prevalence ranged 26.5% to 79%.^44,45^ As opioids are the most prevalent drug in Iran^8^, appropriate strategies and harm reduction programs should be designed to decrease its burden and adverse side effects such as hepatitis C and HIV/AIDS.

Though stimulants marketing is relatively new in Iran, its life time prevalence among those aged 19-29 was estimated at 6%.^24^ Results of a similar study in Tehran confirmed our results.^46^ The lifetime prevalence of stimulants varied among subpopulations, less than 1% among women to more than 13% among body builders.^47^

We have seen remarkable gender difference in terms of opioids and stimulants use. This finding was in line with others results. It is known that men are more likely to show high risk behaviors than women, in particular in Iran due to cultural background.^22,46,48^ Moreover, it has been indicated that men usually transit to drug use from cigarette smoking – prevalence of daily smoking among men is about 24% in Iran – while women initiates with opium.^49^ Despite lower prevalence of opioid use among women, they need specific harm reduction programs because women often provide sexual intercourse in exchange for money or drug. This in turn increases sexual transmitted infections such as HIV/AIDS.^50^

As it had reported in previous works,^9,51^ more educated respondents were less likely to use opioids. It may not be considered as a result of higher awareness or more negative attitude as these were not remained into the final multivariable model.

Those who formed that category “divorced or widow or separated” reported significant higher lifetime prevalence of both of opioid and stimulant use. This population is more likely to have high risk behaviors as compared with single and married populations, partially because of mental distress which is one of the most important reason for initiating drugs.^52,53^ As the number of divorced or separated peoples has been increasing in recent years,^54^ it may be the future concern of the public health in Iran.

## Limitations

We were not able to estimate current prevalence of opioids and stimulants use; as questions about recent use is more sensitive.

We should also mention that we used direct question to estimate prevalence. It has been shown that direct questions prone to prestige biases and might provide an under-estimated prevalence. We already applied indirect methods such as NSU among general population. In NSU, respondents describe their network in terms of drug use. Therefore, prestige bias is of less concern. Instead recall bias and invisibility of hidden characteristics might provide inaccurate estimates.

As no standardized instrument was available, we used a researcher-developed instrument to measure awareness, attitude and life time prevalence. However, this limitation is pervasive in this field in Iran. Therefore, development of a standardized questionnaire for such studies on the drug use would be helpful for future works.

One more limitation of our study was that we were not able to conduct a household survey. We approached pedestrians in the street. Therefore, our sample was not a random sample of the general population. However, our previous experiences suggested that street-based sampling was the optimum approach in the case of sensitive characteristics in Iran.^55,56^

## Conclusion

Despite negative attitudes of Iranian adults toward opioid and stimulant use, their awareness was less adequate to prevent high risk behavior. Men and participants with lower socio-economic status (SES) should be considered as a main target population of health promotion programs for opioid use. However, regarding stimulants use, health promotion programs which focused mostly on youths and who had higher SES could be more effective. Considering wide distribution of virtual world and increasing trend of internet access, we suggest that future studies work on sharing info-graphs of illicit drug use side effects and also assessing educational packages on the network, which could be the fastest, easiest, and most cost-effective way of informing the society. Iran education curriculum needs to be revised in which drug use should be included. In addition, parent should be trained periodically to recognize high-risk behavior warning signs in children and adolescents. Further investigations are needed to identify appropriate and effective approaches based on Iranian socio-cultural context that benefit the population with available resources.

## Acknowledgements

This work has been supported by Iranian Ministry of Health and Medical Education, Tehran, Iran (grant number 93/428).

## Ethical issues

The project was conducted according to the principles of the Helsinki declaration. The anonymity of all participants in each stage of project from focus groups to national respondents remained completely anonymous. The project was approved by Ethical Committee of Kerman University of Medical Sciences, Kerman, Iran (K/93/395).

## Competing interests

Alireza Noroozi, Ahmad Hajebi, and Maryam Mehrabi are affiliated to Ministry of Health and Medical Education (MoHME), Tehran, Iran. But we confirm that there was no competing interest.

## Authors’ contributions

EM, AAH, ARN, AH, RN, AJK, MS, MRB, and MM contributed to the study design. RN performed statistical analyses of national data, interpretation of data, and commenting on manuscript. MRB and EM supervised the study and contributed to all parts of the paper. All authors read and approved the final version of the paper.

## Authors’ affiliations

^1^HIV/STI Surveillance Research Center, and WHO Collaborating Center for HIV Surveillance, Institute for Futures Studies in Health, Kerman University of Medical Sciences, Kerman, Iran. ^2^Modeling in Health Research Center, Institute for Futures Studies in Health, Kerman University of Medical Sciences, Kerman, Iran. ^3^School of Advanced Technologies in Medicine (SATiM), Tehran University of Medical Sciences (TUMS), Tehran, Iran. ^4^Iranian National Center for Addiction Studies (INCAS), Tehran University of Medical Sciences (TUMS), Tehran, Iran. ^5^MPH Department, Medical School, Shiraz University of Medical Sciences, Shiraz, Iran. ^6^Research Center for Addiction & Risky Behavior (ReCARB), Psychiatric Department, Iran University of Medical Sciences, Tehran, Iran. ^7^Department of Sociology, Faculty of Social Sciences and Economics, Alzahra University, Tehran, Iran. ^8^Department of Mental, Social Health and Drug Use, Ministry of Health, Tehran, Iran. ^9^Social Determinants of Health Research Center, Institute for Futures Studies in Health, Kerman University of Medical Sciences, Kerman, Iran.

## Key messages

**Implications for policy makers**

- The life time prevalence of opioids and stimulants were 12.9% (men: 21.5%, women: 4.0%) and 7.3% (men: 9.6%, women: 4.9%) respectively.
- In terms of opioid use, health promotion programs should target men and populations with lower socio-economic status (SES) status.
- Regarding stimulants use, promotion programs should target the younger age groups and those with high SES status.

**Implications for public**

The life time prevalence of opioid and stimulants use among men was 5 and 2 times higher than that of women. Although most Iranians were aware of adverse effects of opioid and stimulants use, the life time prevalence of these outcomes were high. Appropriate health promotion programs should be designed to decrease use of these drugs.
